# A national cross-sectional survey of the attitudes, skills and use of evidence-based practice amongst Spanish osteopaths

**DOI:** 10.1186/s12913-021-06128-6

**Published:** 2021-02-10

**Authors:** Gerard Alvarez, Cristian Justribo, Tobias Sundberg, Oliver P. Thomson, Matthew J. Leach

**Affiliations:** 1 Spain National Centre, Foundation COME Collaboration, Barcelona, Spain; 2 Iberoamerican Cochrane Centre–Biomedical Research Institute Sant Pau, IIB Sant Pau, Barcelona, Spain; 3 Maxilofacial Institute Bara Gaseni - Hospital Sagrado Corazón de Barcelona, Barcelona, Spain; 4 Australian Research Centre in Complementary and Integrative Medicine (ARCCIM), Faculty of Health, University of Technology Sydney, Sydney, NSW Australia; 5 Department of Health Promotion Sciences, Musculoskeletal and Sports Injury Epidemiology Center (MUSIC), Sophiahemmet University & Unit of Intervention and Implementation Research on Worker Health, Institute of Environmental Medicine, Karolinska Institutet, Stockholm, Sweden; 6 University College of Osteopathy, London, UK; 7 Clinical-based Human Research Department, Foundation COME Collaboration, Pescara, Italy; 8 National Centre for Naturopathic Medicine, Southern Cross University, Lismore, NSW Australia

**Keywords:** Evidence-based practice, Osteopathic medicine, Cross-sectional studies, Health care surveys

## Abstract

**Background:**

Although evidence-based practice (EBP) is largely supported across healthcare professions, its implementation in manual therapy professions such as osteopathy remains limited and debated. There is currently little knowledge of how Spanish osteopaths relate to EBP.

**Objectives:**

The main aim of this study was to investigate the attitudes, skills and use of EBP among Spanish osteopaths. A secondary aim was to identify barriers and facilitators for the adoption of EBP in the Spanish osteopathic context.

**Methods:**

National cross-sectional survey of Spanish osteopaths registered and non-registered to an osteopathic association in Spain. Eligible participants were invited by a range of recruitment strategies including email and social media campaigns to complete the Spanish-translated Evidence-Based practice Attitude and utilization Survey (EBASE) anonymously online.

**Results:**

A total of 567 osteopaths completed the survey which represents an approximate response rate of 9%. Participant’s attitudes toward EBP were largely positive. Most respondents agreed or strongly agreed that EBP was necessary in the practice of osteopathy (89.6%) and that professional literature and research findings were useful to their day-to-day practice (88.9%). Levels of perceived skill in EBP were reported as low to moderate with lowest levels for items related to ‘research conduct’. Except reading/reviewing professional literature and using online search engines to find practice-related literature, participant engagement in all other EBP-related activities was generally infrequent. The perceived proportion of clinical practice that was based on clinical research evidence was reported to be very small. Main barriers to EBP uptake included a lack of clinical evidence in osteopathy and insufficient skills for applying research findings. Main facilitators of EBP uptake included access to full-text articles, internet at the workplace and online databases.

**Conclusions:**

Spanish osteopaths were largely supportive of evidence-based practice, had low to moderate skills in EBP and engaged in EBP activities infrequently. Formal regulation of the profession in Spain and the inclusion of osteopathic programs into the university sector would potentially improve EBP skills and use.

**Supplementary Information:**

The online version contains supplementary material available at 10.1186/s12913-021-06128-6.

## Background

Evidence-Based Practice (EBP) has been defined as the integration of knowledge provided by the best available evidence, with a practitioner’s clinical experience and judgement, and combined with an individual patient’s values and preferences [1]. Although there is broad support for EBP across most healthcare professions, its implementation by practitioners of the manual therapy professions remains limited and contested [2–6]. A possible explanation for the limited uptake of EBP in this professional group may be the difficulty in transferring research-based evidence into practice due to the complexity of clinical settings, including the high level of interaction between patients and practitioners [7, 8]. In fact, several healthcare disciplines [9–14] report similar issues in the implementation of EBP, including medicine [14], physical therapy [11] and more recently, osteopathy [15, 16].

Osteopathy is considered a manual therapy discipline in which its therapeutic base has been traditionally embedded within a specific conceptual framework and principles [17–19]. However, several authors have questioned the validity, plausibility and utility of these traditional osteopathic models in the context of contemporary evidence-based healthcare [20–23]. Despite the passing of time, most of these osteopathic models have not been updated in response to the evidence, yielding to an uncritical development of the profession [23]. The lack of integration of EBP in osteopathic professional development has been explored across the world, with the subject receiving significant attention in the UK [24–27].

Osteopathy is defined by The World Health Organisation (WHO) as a first-contact patient-centred health discipline [28]. As osteopathy develops its knowledge base, the profession will need to respond and be critically reflective and move away from the prioritizing of traditional theories and anecdotes as reliable sources of evidence, and instead, pursue the generation of new knowledge through research, and ensure that clinicians and educators are able to integrate evidence into practice. Although it appears that the integration of EBP into osteopathy is largely perceived as a favourable endeavour across the osteopathy community [15, 26, 29], recent findings suggest that osteopathy practitioner engagement with EBP activities seems to be infrequent [26, 30]. Previous research has shown that the perceived barriers to EBP implementation among UK osteopaths are lack of time, lack of evidence in the osteopathic field and lack of access to studies and databases [26]. These results are consistent with a similar previous study conducted in Australia [30].

In Spain, osteopathy is not a regulated healthcare profession [31, 32]. Consequently, osteopaths with different professional, academic and clinical profiles (e.g. qualifications, training standards and competencies) operate across Spain. At the same time, there is considerable heterogeneity across training programs [31, 32]. Notwithstanding, the European Committee for Standardization (CEN) published in 2015 the Standards of Osteopathic Healthcare Provision that specifies, among other requirements, an education, training and ethical framework for the practice of osteopathy [33]. This document highlights *“Scientific rigour and evidenced-informed practice as an important part of osteopathic treatment and patient management”* and includes the *“ability to appraise medical and scientific literature critically and the incorporation of relevant and contemporary information into practice”* as essential competencies for osteopathic practice [33]. In spite of these standards, Spanish osteopath’s attitudes toward, and utilisation of EBP remains largely unknown. After performing a comprehensive literature search on the topic, only one recently published study was identified [34]. While this study provided some insight into the issue in Spain, the methods and selected data extraction tool do not allow direct comparisons to be made with osteopathic communities in other countries. Further exploration of this issue is therefore warranted in order to better inform the development of innovative strategies that might assist Spanish osteopaths to embrace EBP. Responding to this need, the main aim of this study was to investigate the attitudes, skills and use of EBP among Spanish osteopaths. We also aimed to identify the barriers and facilitators for the adoption of EBP in the Spanish osteopathic context.

## Methods

The methods conducted and reported in this present study closely follow those of previously published studies carried out by our group measuring attitudes towards EBP of osteopaths [26, 30, 35] and Chiropractors [36]

### Design

National, online, cross-sectional survey.

### Sample and setting

Due to the unregulated situation of the profession in Spain, the survey was open to all practising osteopaths, regardless of qualification or training institution. Therefore, for the purpose of this study, the term “osteopath/s” applied to any “therapist practicing osteopathy” or any “therapist with training in osteopathy” practising in Spain. No exclusion criteria were applied. Although there is no reliable data on the number of osteopaths practising in Spain, numbers of graduates from osteopathic educational institutions (OEIs) in Spain estimate the Spanish osteopathic workforce to be in the vicinity of 5000–6000 [32]. Based on a target population of 6000, at least 362 osteopaths were required to be surveyed in order to achieve a 5% margin of error and a 95% confidence interval for any individual item on the survey.

### Description of survey and variables

The Evidence-Based practice Attitude and utilization Survey (EBASE) [37] is an 80-item instrument designed to assess attitudes, skills and use of evidence-based practice among health professionals. The survey is divided into seven constructs, including attitude (10 items, rated using a 5-point scale, ranging from “Strongly Agree” to “Strongly Disagree”), skills (13 items, rated using a 5-point scale, ranging from “Low” to “High”), education and training (5 multiple-choice items), use (9 items, rated based on number of articles read/reviewed, number of times performing certain EBP-related activities, and information sources used to inform clinical-decision making), barriers/enablers (23 items, rated using a 4-point scale, ranging from “no barrier / not useful” to “major barrier / very useful”), and demographics (19 multiple-choice items and 1 open-text item). The narrative interpretation of the question, “What percentage of your practice do you estimate is based on clinical research evidence (i.e. evidence from clinical trials)?” was based on the following grading: None (0%); Very small proportion (1–25%); Small proportion (26–50%); Moderate proportion (51–75%); Large proportion (76–99%); or All (100%).

The original English EBASE instrument has demonstrated acceptable test-retest reliability (ICC = 0.578–0.986), good internal consistency (Cronbach’s alpha = 0.84), and good construct and content validity (CVI = 0.899) [37, 38].

Items from three of the survey constructs can be generated into subscores, as follows:

- Attitude subscore: Sum of the first 8 items, with scores ranging from 8 (predominantly strongly disagree) to 40 (predominantly strongly agree).
- Skill subscore: Sum of all 13 items, with scores ranging from 13 (low level skill) to 65 (high level skill).
- Use subscore: Sum of the first 6 items, with scores ranging between 0 (mostly infrequent use) and 24 (mainly frequent use).

### Translation and adaptation of the survey

The questionnaire was translated from English to Spanish and adapted cross-culturally according to the forward and backward translation method recommended by the WHO [39]. Once translated, a pilot test was carried out on 20 purposely-selected Spanish osteopaths. Suggestions for improvement (e.g., provision of additional response options to some questions mainly related to the Spanish educational system) were integrated into the final version of the survey (Additional file 1).

### Recruitment and data collection

Participation in the study was advertised between April 2020 and June 2020 using a range of recruitment strategies. First, the main osteopathic associations (*n* = 9) and OEIs (*n* = 7) in Spain informed their associates (academic/clinical staff and alumni) of the survey and encouraged participation. Second, the study was advertised via social media (Twitter, Facebook and Instagram) inviting osteopaths to complete the EBASE survey. All recruitment media directed participants to a dedicated project website, where participants were invited to read the study information before progressing to the survey. The welcome page contained information on the purpose of the study, the survey constructs, informed consent considerations, reporting of results, storage of data, ethics committee approval and the research team. Once the survey was accessed, participants were provided with instructions on how to complete each section. Those instructions, placed in the beginning of each section, included an explanation on the type of information required and the nature of the response options (Additional file 1).

Data collection was undertaken online using SurveyMonkey™ (SurveyMonkey Inc., San Mateo, California, USA). All survey items were made compulsory to mitigate the risk of missing data.

### Data analysis

Data from the online survey were imported into IBM SPSS version 25 (Armonk, New York, IBM Corp) for data cleaning, coding and statistical analyses. Excluded from the analyses were incomplete surveys (i.e. surveys containing > 20% missing data due to participant dropout) and duplicate responses (i.e. as identified using the Konstan et al. de-duplication procedure for online surveys [40]). Categorical data were described using frequencies and percentages. Means and standard deviations were used to describe normally distributed data, and medians and the interquartile range used for non-normally distributed data. Associations between ordinal-level variables were examined using the Kendall’s Tau correlation coefficient (Ƭ), whereas relationships between nominal-level variables were assessed using Cramer’s V. Coefficients were interpreted as follows: 0.10–0.29 (weak association), 0.30–0.49 (moderate association) and 0.50–1.00 (strong association) [41]. The level of significance was set at *p* < 0.05. All correlations were determined a priori (based on analyses reported for other studies using EBASE). To ensure comparability across studies using EBASE, analyses were not corrected for multiple comparisons.

### Ethics

The study was approved by the Ethical Committee of Sagrado Corazón Hospital, Barcelona (2019/81-CMF-HUSC). Study participation was anonymous and voluntary, and participants were able to withdraw from the survey at any time without repercussion. Informed consent was obtained online from all subjects and all methods were carried out in accordance with relevant guidelines and regulations.

## Results

The survey was commenced by 714 Spanish osteopaths. After removing duplicate entries (*n* = 4) and incomplete responses (*n* = 143), the adjusted sample size was 567. The reasons for participant dropout could not be examined due to the anonymous nature of the survey. It also was not possible to calculate a survey response rate as the actual reach of the survey could not be determined. However, considering a potential target population of 6000 Spanish osteopaths [32] the estimated response rate would be approximately 9% (564/6000).

### Characteristics of sample

**Fig. 1:**
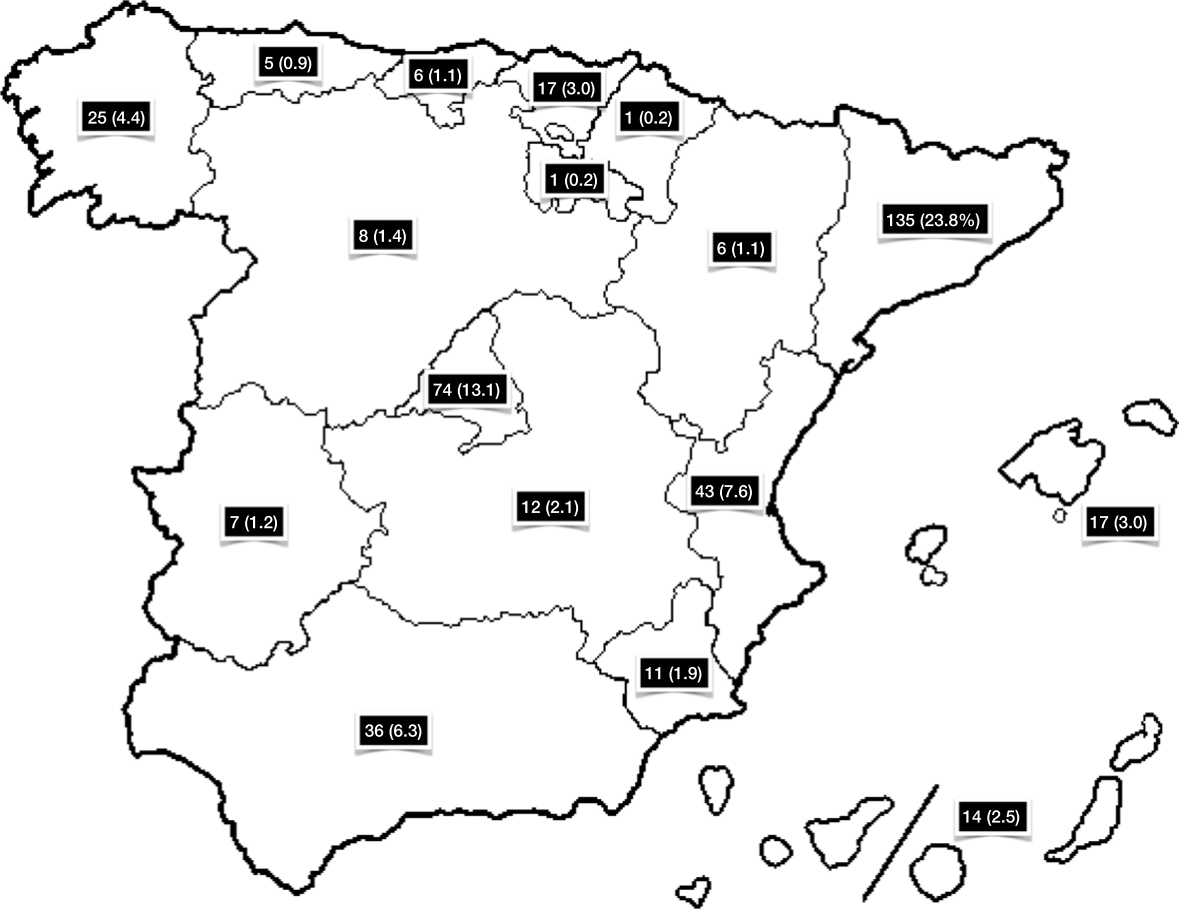
Geographical location of practice, n (%)

Participating osteopaths were predominantly male (47.1%), and aged between 30 and 49 years (62.6%); 40.7% were members of an osteopathy professional association (Table 1). The largest proportion of participants held a Master’s degree as their highest qualification (37.9%), with 50.5% receiving this qualification within the past 10 years. Two-thirds (66.0%) of participants also held a degree in physiotherapy. Most (59.5%) participants had been in osteopathic practice for six or more years, with 57.7% spending 16–45 h/week in clinical practice, 72.3% spending up to 15 h/week participating in research, and 50.4% working up to 15 h/week teaching in the higher education sector. Participating osteopaths largely worked in solo practice (41.4%) in the central business district (43.4%), with most located in Cataluña (23.8%), Comunidad de Madrid (13.1%) or Comunidad Valenciana (7.6%) (Fig. 1). The missing values reported in the results represent participants that did not answer the question as they had withdrawn from the survey at that point.

**Table 1:** Demographic characteristics of sample (*n* = 567)

Characteristic	Frequency, n (%)
**Age**, n (%)	
18–20 years	3 (0.5)
20–29 years	23 (4.1)
30–39 years	187 (33.0)
40–49 years	168 (29.6)
50–59 years	30 (5.3)
60–69 years	10 (1.8)
70+ years	1 (0.2)
Missing	145 (25.6)
**Sex**, n (%)	
Male	267 (47.1)
Female	155 (27.3)
Missing	145 (25.6)
**Osteopathy professional association membership**, n (%)	
Not a member of an Osteopathy professional association	185 (32.6)
Registry of Osteopaths of Spain	103 (18.2)
Registry of Osteopathic Physiotherapists of Spain	85 (15.0)
Other Osteopathy professional association	43 (7.6)
Missing	151 (26.6)
**Highest qualification**, n (%)	
Diploma	82 (14.5)
Bachelor degree	27 (4.8)
Master’s degree	215 (37.9)
PhD	46 (8.1)
Other	52 (9.2)
Missing	145 (25.6)
**Years since receiving highest qualification**, n (%)	
< 1 year	42 (7.4)
1–5 years	150 (26.5)
6–10 years	94 (16.6)
11–15 years	59 (10.4)
16+ years	76 (13.4)
Missing	146 (25.7)
**Other non-osteopathic qualifications**, n (%)	
Degree in Physiotherapy	374 (66.0)
Diploma/Degree in other health-related field	31 (5.5)
Degree in Medicine	5 (0.9)
Degree in Nursing or Podiatry	5 (0.9)
Missing	152 (26.8)
**Years practiced in the field of osteopathy**, n (%)	
< 1 year	6 (1.1)
1–5 years	78 (13.8)
6–10 years	136 (24.0)
11–15 years	95 (16.8)
16+ years	16 (18.7)
Missing	146 (25.7)
**Hours per week in clinical (osteopathic) practice**, n (%)	
0 h	5 (0.9)
1–15 h	47 (8.3)
16–30 h	150 (26.5)
31–45 h	177 (31.2)
46+ hours	42 (7.4)
Missing	146 (25.7)
**Hours per week participating in research**, n (%)	
0 h	162 (28.6)
1–15 h	248 (43.7)
16–30 h	9 (1.6)
31–45 h	2 (0.4)
46+ hours	0 (0.0)
Missing	146 (25.7)
**Hours per week working in the higher education sector**, n (%)	
0 h	189 (33.3)
1–15 h	97 (17.1)
16–30 h	59 (10.4)
31–45 h	29 (5.1)
46+ hours	47 (8.3)
Missing	146 (25.7)
**Clinical setting in which osteopathy was predominantly practiced**, n (%)	
Solo practice	235 (41.4)
With a group of allied health providers	87 (15.3)
With a group of complementary and allied health providers	41 (7.2)
With conventional and allied health providers	37 (6.5)
Within a clinical institution (e.g. hospital)	12 (2.1)
Within an educational institution (e.g. university)	6 (1.1)
Missing	149 (26.3)
**Geographical region of practice**, n (%)	
City (Central business district)	246 (43.4)
Inner city location	88 (15.5)
Rural location	43 (7.6)
Outer city location	39 (6.9)
Missing	151 (26.6)

*CAM* Complementary and alternative medicine

### Use of EBP

Participants reported a moderate-low level of engagement in EBP activities (median use subscore 8; IQR 5,14; range 0–24; scores ranging between 6.1 and 12.0 are indicative of a moderately-low level of use). Reading/reviewing professional literature and using online search engines to find practice-related literature were among the most frequently reported activities, with 44.6–48.0% of participating osteopaths engaging in these activities between 1 and 10 times in the month preceding the survey (Table 2). Participant engagement in all other EBP-related activities was generally infrequent, with 49.6–67.2% of participating osteopaths engaging in these activities no more than 5 times in the previous month.

**Table 2:** Participant engagement in evidence-based practice activities within the last month (*n* = 567)

	***0*** 0 times ***n (%)***	***1*** 1–5 times ***n (%)***	***2*** 6–10 times ***n (%)***	***3*** 11–15 times ***n (%)***	***4*** 16+ times ***n (%)***	Missing ***n (%)***	Median (IQR)
**I have used an online search engine to search for practice related literature or research**	65 (11.5)	157 (27.7)	96 (16.9)	51 (9.0)	117 (20.6)	81 (14.3)	3 (2,4)
**I have read/reviewed professional literature (i.e. professional journals & textbooks) related to my practice**	65 (11.5)	184 (32.5)	88 (15.5)	46 (8.1)	107 (18.9)	77 (13.6)	2 (2,4)
**I have used an online database to search for practice related literature or research**	107 (18.9)	174 (30.7)	66 (11.6)	36 (6.3)	103 (18.2)	81 (14.3)	2 (2,4)
**I have used professional literature or research findings to assist my clinical decision-making**	101 (17.8)	215 (37.9)	71 (12.5)	40 (7.1)	63 (11.1)	77 (13.6)	2 (2,3)
**I have used professional literature or research findings to change my clinical practice**	110 (19.4)	235 (41.4)	58 (10.2)	29 (5.1)	58 (10.2)	77 (13.6)	2 (2,3)
**I have read/reviewed clinical research findings related to my practice**	125 (22.0)	193 (34.0)	62 (10.9)	35 (6.2)	75 (13.2)	77 (13.6)	2 (1,3)
**I have consulted a colleague or industry expert to assist my clinical decision-making**	113 (19.9)	220 (38.8)	67 (11.8)	33 (5.8)	53 (9.3)	81 (14.3)	2 (2,3)
**I have referred to magazines, layperson / self-help books, or non-government/non-education institution websites to assist my clinical decision-making**	236 (41.6)	145 (25.6)	39 (6.9)	25 (4.4)	41 (7.2)	81 (14.3)	2 (1,2)

*IQR* Interquartile range

A weak positive association was found between use subscore (categorised by quartiles) and age (Ƭ = .113, *p* = .007), sex (V = .155, *p* = .018; with higher subscores reported among male participants), highest qualification (Ƭ = .165, *p* < .001), years in osteopathy practice (Ƭ = .142, *p* < .001), number of hours in clinical practice (Ƭ = .082, *p* = .037), and number of hours teaching in the higher education sector (Ƭ = .225, *p* < .001). A moderate positive association was observed between use subscore and number of hours engaged in research work (Ƭ = .345, *p* < .001).

The perceived proportion of clinical practice that was based on clinical research evidence was reported to be very small by 21.5% of participants, small by 21.7% of participants, moderate by 26.8% of participants and large by 12.0% of participants. Few participants reported that none or all of their practice was underpinned by clinical research evidence.

The information sources used most frequently by participating osteopaths to inform their clinical decision-making were traditional knowledge (median rank 4; IQR 2,7) and published clinical evidence (median rank 4; IQR 2,7) (Table 3: ranked from 1 = most frequently used, to 10 = least frequently used). Published experimental/laboratory evidence was used by participants the least (median rank 8; IQR 3,10).

**Table 3:** Sources of information used by participants to inform their clinical decision-making (in descending order of frequency) (*n*=567)

Information source	Median (IQR)
Traditional knowledge	4 (2,7)
Published clinical evidence (i.e. clinical trials)	4 (2,7)
Consulting fellow practitioners or experts	4 (3,6)
Textbooks	5 (2,7)
Personal intuition	5 (3,7)
Clinical practice guidelines	6 (3,8)
Personal preference	7 (4,9)
Trial and error	7 (4,9)
Patient preference	7 (5,9)
Published experimental/laboratory evidence	8 (3,10)

*IQR* Interquartile range

### Skills and training in EBP

Overall, participating osteopaths reported a low-moderate to moderate level of perceived skill in EBP (median skill subscore 39; IQR 31,48; range 13–65; scores ranging between 26.1 and 39.0 are indicative of a low-moderate to moderate skill level). Relatively higher levels of perceived skill were observed for items pertaining to ‘clinical problem identification’ (i.e. identifying answerable clinical questions [73.6% self-reported a moderate to moderate-high skill level for this item], and identifying knowledge gaps in practice [68.5% self-reported a moderate to moderate-high skill level for this item]) (Table 4). Participants reported the lowest levels of perceived skill for items related to ‘research conduct’, with more than one-half of participating osteopaths reporting low to low-moderate skill in conducting clinical research (60.4%) and systematic reviews (50.9%).

**Table 4:** Participant’s perceived skill level in evidence-based practice (*n* = 567)

	***1*** Low ***n (%)***	***2*** Low-moderate ***n (%)***	***3*** Moderate ***n (%)***	***4*** Moderate-high ***n (%)***	***5*** High ***n (%)***	Missing ***n (%)***	Median (IQR)
**Identifying answerable clinical questions**	14 (2.5)	56 (9.9)	183 (32.3)	234 (41.3)	58 (10.2)	22 (3.9)	4 (3,4)
**Identifying knowledge gaps in practice**	25 (4.4)	84 (14.8)	222 (39.2)	166 (29.3)	48 (8.5)	22 (3.9)	3 (3,4)
**Locating professional literature**	38 (6.7)	102 (18.0)	148 (26.1)	174 (30.7)	83 (14.6)	22 (3.9)	3 (2,4)
**Online database searching**	44 (7.8)	101 (17.8)	143 (25.2)	155 (27.3)	102 (18.0)	22 (3.9)	3 (2,4)
**Retrieving evidence**	46 (8.1)	112 (19.8)	158 (27.9)	145 (25.6)	84 (14.8)	22 (3.9)	3 (2,4)
**Critical appraisal of evidence**	33 (5.8)	122 (21.5)	190 (33.5)	143 (25.2)	43 (7.6)	36 (6.3)	3 (2,4)
**Synthesis of research evidence**	28 (4.9)	121 (21.3)	185 (32.6)	150 (26.5)	47 (8.3)	22 (3.9)	3 (2,4)
**Applying research evidence to patient cases**	25 (4.4)	74 (13.1)	185 (32.6)	197 (34.7)	50 (8.8)	36 (6.3)	3 (3,4)
**Sharing evidence with colleagues**	53 (9.3)	100 (17.6)	139 (24.5)	159 (28.0)	80 (14.1)	36 (6.3)	3 (2,4)
**Using findings from clinical research**	73 (12.9)	119 (21.0)	169 (29.8)	127 (22.4)	39 (6.9)	40 (7.1)	3 (2,4)
**Using findings from systematic reviews**	89 (15.7)	131 (23.1)	127 (22.4)	136 (24.0)	44 (7.8)	40 (7.1)	3 (2,4)
**Conducting systematic reviews**	147 (25.9)	142 (25.0)	122 (21.5)	82 (14.5)	34 (6.0)	40 (7.1)	2 (1,3)
**Conducting clinical research**	218 (38.4)	125 (22.0)	102 (18.0)	53 (9.3)	33 (5.8)	36 (6.3)	2 (1,3)

*IQR* Interquartile range

A weak positive association was found between skill subscore (categorised by quartiles) and age (Ƭ = .140, *p* = .001), sex (V = .256, *p* < .001; with higher subscores reported among male participants), highest qualification (Ƭ = .254, *p* < .001), years in osteopathy practice (Ƭ = .227, *p* < .001), number of hours in clinical practice (Ƭ = .086, *p* = .033), and number of hours teaching in the higher education sector (Ƭ = .265, *p* < .001). Skill subscore was shown to be moderately positively correlated with number of hours engaged in research work (Ƭ = .437, *p* < .001).

The majority (> 62%) of participating osteopaths had completed training in EBP, evidence application, conducting clinical research, conducting systematic reviews and critical thinking. Across all five areas, training was mostly undertaken as a minor or major component of a university course (i.e. by 24.3 to 37.7% of participants), or as a short course (i.e. by 9.2 to 17.8% of participants).

### Attitudes toward EBP

Participants’ attitudes toward EBP were largely positive (median attitude subscore 32; IQR 28,35; range 11–40; scores ranging between 32.0 and 40.0 are indicative of a predominantly agree to strongly agree response). At least 4 out of 5 participating osteopaths agreed or strongly agreed that EBP was necessary in the practice of osteopathy (89.6%) and professional literature and research findings were useful to their day-to-day practice (88.9%), with a similar proportion expressing an interest in learning or improving the skills necessary to incorporate EBP into their practice (88.9%) (Table 5). On the other hand, a large proportion (43.5%) of participants disagreed or strongly disagreed that EBP placed an unreasonable demand on their practice.

**Table 5:** Participant attitudes toward evidence-based practice (*n* = 567)

	***1*** Strongly Disagree ***n (%)***	***2*** Disagree ***n (%)***	***3*** Neutral ***n (%)***	***4*** Agree ***n (%)***	***5*** Strongly Agree ***n (%)***	Median (IQR)
**I am interested in learning or improving the skills necessary to incorporate EBP into my practice**	12 (2.1)	16 (2.8)	35 (6.2)	202 (35.6)	302 (53.3)	5 (4,5)
**EBP is necessary in the practice of osteopathy**	14 (2.5)	17 (3.0)	28 (4.9)	225 (39.7)	283 (49.9)	4 (4,5)
**Professional literature (i.e. journals & textbooks) and research findings are useful in my day-to-day practice**	10 (1.8)	13 (2.3)	40 (7.1)	288 (50.8)	216 (38.1)	4 (4,5)
**EBP improves the quality of my patient’s care**	17 (3.0)	27 (4.8)	56 (9.9)	234 (41.3)	233 (41.1)	4 (4,5)
**EBP assists me in making decisions about patient care**	12 (2.1)	30 (5.3)	63 (11.1)	260 (45.9)	202 (35.6)	4 (4,5)
**Prioritizing EBP within osteopathic practice is fundamental to the advancement of the profession**	21 (3.7)	48 (8.5)	63 (11.1)	211 (37.2)	224 (39.5)	4 (4,5)
**EBP takes into account my clinical experience when making clinical decisions**	21 (3.7)	73 (12.9)	98 (17.3)	214 (37.7)	161 (28.4)	4 (3,5)
**EBP takes into account a patient’s preference for treatment**	36 (6.3)	143 (25.2)	116 (20.5)	173 (30.5)	99 (17.5)	3 (2,4)
**There is a lack of evidence from clinical trials to support most of the treatments I use in my practice**	41 (7.2)	138 (24.3)	151 (26.6)	178 (31.4)	59 (10.4)	3 (2,4)
**The adoption of EBP places an unreasonable demand on my practice**	50 (8.8)	197 (34.7)	148 (26.1)	141 (24.9)	31 (5.5)	3 (2,4)

*EBP* Evidence-based practice, *IQR* Interquartile range

The attitude subscore (categorised by quartiles) was found to be weakly positively associated with participant sex (V = .162, *p* = .011; with more favourable attitudes reported among male participants), number of hours teaching in the higher education sector (Ƭ = .104, *p* = .013), and number of hours engaged in research work (Ƭ = .133, *p* = .003).

A secondary analysis was performed to assess the relationship between membership of a professional association and attitude, skill and use subscores. Our results showed a weak positive association between professional association membership and skill subscore (V = .130, *p* = .031; i.e. higher perceived skill subscore among those reporting membership of a professional association) and use subscore (V = .170, *p* = .003; i.e. higher use subscore among those reporting membership of a professional association). In a further analysis between associations, we found a weak positive association between professional association affiliation and skill subscore (V = .213, *p* < .001; i.e. higher perceived skill subscore among those reporting membership of ROFE) and use subscore (V = .168, *p* = .002; i.e. higher use subscore among those reporting membership of ROFE).

### Barriers and enablers of EBP use

Of the 13 listed barriers to EBP uptake, participants identified 8 as being minor to moderate barriers, including lack of clinical evidence in osteopathy (51.3%), insufficient skills for applying research findings (50.8%), lack of time (49.9%), insufficient skills for appraising research (48.8%), insufficient skills for interpreting research (47.1%), insufficient skills for locating research (46.0%), lack of industry support for EBP (45.1%), and lack of incentive to participate in EBP (44.4%). The remaining 5 barriers (i.e. lack of resources, interest, relevance, colleague support, and patient preference) were considered by many (49.2 to 63.1%) participants as not being a barrier or only a minor barrier to EBP uptake.

Most participating osteopaths indicated that the 10 listed enablers of EBP uptake were either moderately or very useful. These enablers included improving access to: full-text journal articles (63.8% rated this strategy as moderately or very useful), internet in the workplace (63.3%), free online databases (63.0%), online EBP education materials (62.8%), critical reviews of research evidence relating to osteopathy (61.0%), research rating tools (58.2%), online tools that facilitate practitioner appraisal of the evidence (58.0%), databases requiring licence fees (57.3%), critically appraised topics relating to osteopathy (57.3%), and critical appraisal tools (56.3%).

## Discussion

This study aimed to assess the attitudes, skills and utilisation of EBP, as well as the barriers and enablers of EBP use, among Spanish osteopaths. Overall, our results showed that Spanish osteopaths hold positive views towards EBP but their utilization/implementation is still poor. Positive responses about EBP training and utilisation were modest and, in general, the perceived level of EBP-related skill was very low. Despite these low levels of skill in relation to the utilization of EBP, several barriers to the use of EBP were perceived as not important by participants. Although it was not possible to calculate the mean response and the number of withdrawals from the survey, the final number of participants that completed the survey was around 9% of the target population, which is similar to the survey response rate reported for osteopaths in a similar UK study [26]

Our study sample had a high proportion of male participants and those aged between 30 and 49 years, which was similar to that reported in another recent survey of Spanish osteopaths [32]. Previous studies in the UK and Australia have also reported comparable gender distributions [26, 30] although in Sweden this distribution was more balanced [35]. In our survey, 73% of osteopaths had a previous degree in a healthcare profession regulated in Spain. This is consistent with other surveys conducted in Spain [32], where the majority of osteopaths have reported a previous degree in physiotherapy and postgraduate training in osteopathy.

### EBP attitudes

Most participating Spanish osteopaths agreed that EBP is necessary in the practice of osteopathy, with the majority interested in learning or improving the skills necessary to incorporate EBP into their practice. Overall, attitudes toward EBP were positive which is in accordance with a recent study also conducted in Spain [34]. In fact, this favourable view of EBP has been also reported in other healthcare professions, such as nursing [42, 43], physiotherapy [11, 44, 45], dentistry [46] and chiropractic [12], as well as osteopaths in other countries [26, 30, 35].

Although attitudes towards EBP were generally positive, a considerable number of respondents (30%) believed that EBP placed an unreasonable demand on their practice. This item was much less reported by Australian (17%) [30], UK (16%) [26] and Swedish osteopaths (17%) [35]. This suggests that almost one-third of our sample may be experiencing difficulties in integrating EBP into their daily practice. Therefore, further efforts are required to enhance the competence, skills and efficiency of implementing the components of EBP amongst osteopaths in Spain. A possible explanation for these contrasting findings could be the lack of professional regulation in Spain, even compared to osteopathy in Sweden, where like Spain, osteopathy is also an emerging profession. However, in Sweden, there are far fewer OEIs (currently just one), and just a single osteopathic association of which the majority of osteopaths are members of [35]. The relatively higher number of OEIs in Spain and the diversity of educational focus (such as time spent on EBP) may explain the burden that Spanish osteopaths perceive in enacting EBP.

Research from nursing and physical therapy has found the level of professional education to be predictive of the propensity to adopt EBP; although, other factors were also influential, such as desire to learn, practicality (i.e. non-interference with productivity or patient flow) and beliefs about EBP [47, 48]. Indeed, there was some indication in our study that participating osteopaths had a misconstrued understanding of the EBP paradigm, with a large proportion of participants believing that EBP did not take into consideration the patient’s perspective. Other studies have also indicated that osteopaths view EBP as a threat to the professional identity of the profession [24, 25, 49]. This suggests that Spanish osteopaths might benefit from revisiting the fundamental purposes of contemporary EBP which seeks to prioritise ethical care, is relationship-based, advocates shared decision-making and relates the evidence to the individual patient’s context and situation [7].

### EBP skills

Participating Spanish osteopaths perceived their EBP skill level to be low to moderate, which is relatively lower than that reported among osteopaths in other countries [26, 30, 35], chiropractors [12] and physiotherapists [44]. A notable insecurity among participants related to the conduct of clinical studies or systematic reviews. This finding was somewhat expected as these advanced research activities are not typically taught within undergraduate clinical programs.

Another self-reported skill-deficit related to the implementation of research findings, that is, the process of applying research evidence into clinical practice. This coincides with the perceived barriers of EBP uptake reported by participants, of which half of our sample identified insufficient skills in applying research findings as a barrier to EBP utilisation. In fact, this is a problem affecting many healthcare professions, where considerable ‘research-practice gaps’ exist [50–52]. However, these research-practice gaps can be attributed to more than just insufficient knowledge and skills; organizational factors, and social and attitudinal behaviours are also important contributors [50]. We argue that all of these factors may be in part responsible for the limited application of research evidence in Spanish osteopathic practice given the historical lack of research culture within the profession in both the clinical and academic domain.

The impact of research culture on EBP use was supported to some extent by the association between EBP use and number of hours engaged in research work and teaching in the higher education sector. Although this association has been already reported amongst Spanish osteopaths [34], when this finding is compared against surveys of Australian [30] or UK [26] osteopaths, there are similarities in the number of hours dedicated to research activities (i.e. 0–15 h/week; Spain = 72%, Australia = 84%, UK = 72%), though Spanish osteopaths did seem to dedicate more hours to teaching in the higher education sector (i.e. 1–30 h/week; Spain = 28%, Australia = 15%, UK = 18%). Hence, one might expect Spanish osteopaths to have a higher perceived level of EBP-related skill, although this was not the case. This raises questions about the integration of EBP and research into Spanish osteopathic curricula.

### Use of EBP

A moderately-low level of engagement in EBP activities was reported in our sample. An interesting finding was the relatively high proportion of participants that had not searched an online database (18.9%) or used scientific literature to change their clinical practice (19,4%) in the month preceding the survey. This behaviour can be partially explained by the types of information sources used by our sample. The main source of information used by participants to inform their clinical decision-making was traditional knowledge, followed by published clinical evidence and peer opinion or textbooks. These results highlight the importance of traditional knowledge to Spanish osteopaths; a finding that also can be extended to osteopaths in Australia [30], UK [26] and Sweden [35], who also report traditional knowledge as the primary source of information used to inform clinical decision-making. However, while Australian and UK osteopaths cited clinical guidelines as their second most frequently used information source, in the present study, Spanish osteopaths rated these guidelines as their sixth most frequently used source of information and in comparison, it was reported in ninth position by Swedish osteopaths [35]. Given that clinical guidelines typically represent the best available evidence in a field, a considerable research-practice gap may be evident in Spanish and Swedish osteopathic practice.

Notwithstanding, there appear to be few differences in EBP use between these countries. Similar to our sample, the use of EBP among osteopaths in Australia, UK and Sweden is reported to be generally low [26, 30, 35], as it is among US and Canadian chiropractors [9, 53]. Existing evidence suggests physical therapists have a relatively greater level of engagement with EBP [44, 54]. Interestingly, two-thirds of our sample held a qualification in physiotherapy, which is common among osteopaths in Spain [31, 32]. This result raises the question as to why participating osteopaths engage with EBP to a lesser extent than physiotherapists. One explanation is that to date, we have no data on Spanish physiotherapist engagement in EBP activities, which possibly could be different from other countries. Further, almost half of our sample received their highest qualification at least 6 years before the survey, and in Spain, EBP has been only recently integrated into physiotherapy curricular [55]. Osteopathy also has been traditionally embedded in clinical models and frameworks conceived by a small number of individuals from when the discipline was attempting to establish itself, leading to an uncritical development of the profession [23]. An examination of the comparative uptake of EBP among different health disciplines in Spain may shed some further light on this issue.

In terms of potential solutions to improving EBP uptake, education about EBP alone may not be sufficient [56]. Findings from a recent scoping review suggest education programs aimed at improving local barriers may be a more effective method of improving EBP engagement [57]. Within osteopathy, these barriers to EBP uptake are not entirely clear. Indeed, in the case of our respondents, most did not perceive there to be any major barriers.

### Barriers and enablers for EBP

Previous surveys of osteopaths [26, 30, 35] have reported lack of clinical evidence in osteopathy (Australia = 60%; UK = 69%; Sweden = 53%) and lack of time (Australia = 53%; UK = 57%) as major barriers to EBP uptake. Although none of the thirteen listed factors in the survey were identified as major barriers to EBP uptake among Spanish participants, lack of clinical evidence in osteopathy and lack of time were still considered the most notable issues of concern, with at least one-half of respondents reporting these as minor to moderate barriers. Despite the rather high proportion of academics noticed in our sample and in another study conducted in Spain [34], the main osteopathic profile in this country is clinical-based with the 60% of osteopaths dedicating 5 days per week to clinical practice [32]. This scenario could explain the time constraints to be involved in research activities.

Other important barriers related to lack of skills (applying research, research appraisal and interpreting research), showing the necessity to improve these abilities in order to encourage EBP uptake among new osteopaths. While education is important, there also needs to be a change in mindset, triggered by a comprehensive reflection that considers osteopathy as a healthcare profession that embraces science as a way to evolve and self-assess [49].

Exactly how EBP and research culture should be promoted among healthcare practitioners remains controversial. Several recent scoping reviews in the field of physiotherapy suggests some ideas [57, 58]. The first consideration is that any strategy aimed at improving EBP uptake should be tailored to the local scenario to contextualize the training program. The second consideration is that multifaceted strategies (containing at least five elements) are more likely to be associated with significant changes in learning outcomes. Stander et al. [57] also highlights the need for EBP training programmes to be underpinned by behaviour change models, learning theories and a concrete theoretical framework. A recent systematic review and Delphi survey proposes a set of 68 core competencies as a possible framework to inform the development of EBP curricula for health professionals [59].

Regarding enablers, around 63% of participants considered free access to scientific evidence, access to the internet at work, access to free databases and online EBP education materials to be very useful enablers. This was in line with osteopath’s reports in other countries [26, 30, 35]. In the case of Spain, we hypothesize that the national unification of osteopathic curricula and the inclusion of these programmes in universities may also contribute to improvements in EBP skills and uptake. In fact, a higher academic degree has been shown to be associated with a higher propensity to adopt EBP and this has also been observed in Spanish osteopaths [34]. Furthermore, educational programmes and their associated curricula can act as a key driver for shaping healthcare professionals’ knowledge, skills and attitudes towards activities associated with EBP [60]. Not surprisingly, integrating EBP into the curricula of academic programs has become an essential requirement for academic institutions providing training for healthcare professions [61–63].

But above all, we consider that it is necessary to change the way EBP is taught within osteopathic educational programs in Spain. Currently, for many OEIs, EBP is incorporated within the curriculum by teaching specific research techniques, methods and statistics in the final years of training. Instead, we propose that the philosophy of EBP should be promoted from the beginning and embedded along the whole programme. Such integration should be facilitated by the explicit inclusion of EBP as a core competency within professional standards and requirements in addition to accreditation processes [60]. OEIs directors should commit to design their programmes according to this perspective and teachers should present their contents always balancing the knowledge acquired from tradition and experience with the knowledge obtained from research literature and adequate appraisal. In summary, we argue that a better professional qualification of osteopaths is necessary in Spain and that this process must be based on a pedagogical shift from the mere delivery of contents to the promotion of critical thinking and reflective practice. These actions will potentially improve the consistency of programmes, ensure course content represents best practice, and help nurture a stronger research and EBP culture within the profession. While these actions might be difficult to accomplish in Spain without national professional regulation, results from Sweden (where osteopathy is also an unlicensed health profession) demonstrate that extensive exposure to EBP training (91% of Swedish osteopaths vs. 62% of Spanish osteopaths) correlates with higher perceived skills in EBP [35].

### Limitations

Although the survey response rate exceeded the minimum sample size required, we noticed a high percentage of missing responses. As all responses were compulsory, this meant many respondents dropped out of the survey early, possibly due to survey fatigue. This was also a limitation that was discussed in a UK study of osteopaths [26]. Nonetheless, as the original English version of EBASE has demonstrated good internal consistency and acceptable test-retest reliability [37, 38], and the fact that survey fatigue was not detected during pilot testing, it was not considered appropriate to modify the number of survey items.

While the process of translating the questionnaire strictly followed standard methods reported by WHO [39], including a pilot study, the psychometric properties of the Spanish version of the questionnaire was not formally assessed, which could have impacted the validity and reliability of findings. As has occurred in other osteopathic surveys conducted in Spain [31, 32], a large proportion of responses came from two geographical regions (Cataluña and Madrid). These results likely reflect the geographical distribution of osteopaths in Spain. Notwithstanding, there were a number of regions that were not well represented, whose views might potentially differ from the more well-represented regions. Recall bias and selection bias are other intrinsic limitations of the study design.

## Conclusions

This study furthers our understanding of Spanish osteopaths’ attitudes, skills and use of EBP. Our results showed that participant’s attitudes toward EBP were largely positive. At least four out of five participating osteopaths agreed that EBP was necessary in the practice of osteopathy and professional literature and research findings were useful to their day-to-day practice. However, the perceived skills, use and engagement in EBP activities was reported as moderate to low. Fortunately, there was general interest in learning and improving the skills necessary to incorporate EBP into clinical practice. Our findings, therefore, suggest that wider inclusion of osteopathic educational programmes into university environments along with a pedagogical shift towards the promotion of critical thinking and reflective practice may be helpful in assisting Spanish osteopaths to embrace EBP. Although, as discussed in this paper, the absence of osteopathy regulation in Spain could potentially continue to curtail efforts to improve EBP uptake in this professional group.

## Supplementary Information

**Additional file 1.**

## Data Availability

The datasets used and/or analysed during the current study are available from the corresponding author on reasonable request.

## Abbreviations

EBP: Evidence-Based Practice
WHO: World Health Organization
OEIs: Osteopathic Educational Institutions
EBASE: Evidence-Based practice Attitude and Utilization Survey
CAM: Complementary and Alternative Medicine
