# Supplementary material for: A national cross-sectional survey of the attitudes, skills and use of evidence-based practice amongst Spanish osteopaths
Source: BMC Health Serv Res. 2021 Feb 10;21:130. doi: 10.1186/s12913-021-06128-6 (PMC7874623; doi:10.1186/s12913-021-06128-6)
Supplement: Supplementary file 1 — Additional file 1. [file 12913_2021_6128_MOESM1_ESM.pdf]

INNOVATE-UK: International Osteopathy and Evidence-Based Practice Project

Welcome

Evidence-based practice (EBP) is described as a problem-solving framework that facilitates the use of the best available evidence to make decisions about the care of individual patients. Although the use of EBP amongst all health professions is encouraged, we do not yet have a good understanding of osteopath's use of EBP. This study serves to address this need.

If you are an osteopath, we invite you to participate in this brief (10-minute) survey to explore osteopath use, opinion, skills and training in EBP, as well as the barriers and enablers of EBP use, in order to better understand the factors that may influence an osteopath's uptake of EBP.

By completing this survey you are consenting to participation in this study; however, your participation is voluntary and you can withdraw from the study at any time. Your participation and/or withdrawal from this study will have no impact on your relationship with the researchers or their affiliated institutions. You also have the right to ask any questions about the study at any time. So please contact Dr Oliver Thomson as per the contact information below if you have any questions or concerns relating to this study.

Please note that all information collected will remain confidential, and that the data obtained will not be used for any other purpose except for this study. Findings from the study may be published in a peer-reviewed journal, disseminated to professional manual therapy associations, and presented at international conferences and research seminars. A summary of the research findings also will be available to participants (upon request) from 1st August 2017. While your responses will be kept confidential by the researchers and not be identified in the reporting of this research, the researcher cannot guarantee the confidentiality or anonymity of material transferred by email or the internet.

This study has been approved by the British School of Osteopathy Research Ethics Committee. Any ethical concerns relating to the conduct of this study can be addressed to the Secretary of the British School of Osteopathy Research Ethics Committee, Dr Mike Ford, on 0207 089 5330 or m.ford@bso.ac.uk.

Sincerely,

Dr Oliver Thomson
Senior Lecturer | British School of Osteopathy
275 Borough High Street, London, SE1 1JE
Tel: 020 70895330 | Email: o.thomson@bso.ac.uk

INNOVATE Project Team:
Dr. Oliver Thomson | BSc(Hons), DO, MSc, PhD | British School of Osteopathy, United Kingdom
Dr. Michael Ford | PhD | British School of Osteopathy, United Kingdom
Dr. Tobias Sundberg | PT, DO, BScMed, MScMed, PhD | Karolinska Institutet, Sweden
Dr. Matthew Leach | RN, BN(Hons), ND, PhD | University of South Australia, Australia
Prof. Jon Adams | BA(Hons), MA, PhD | University of Technology Sydney, Australia
Dr. Phil Austin | BSc(Osteopathy), MSc, PhD | Pallister House, Greenwich, Australia

*** 1. Before proceeding, please indicate below whether you agree with the following statement:**

- **I am a registered osteopath practicing in the United Kingdom,**
- **I understand what participation in this study entails, and**
- **I know what my rights are as a participant in this study**

○ I agree

○ I disagree

EBASE

INNOVATE-UK: International Osteopathy and Evidence-Based Practice Project

PART A: Beliefs

Part A of the survey asks you to rate your opinion on some statements relating to evidence-based practice; the scale ranges from strongly disagree to strongly agree (Please try to avoid answering 'neutral' unless you really are uncertain)

*** 2. Evidence-based practice is necessary in the practice of osteopathy**

Strongly Disagree	Disagree	Neutral	Agree	Strongly Agree
○	○	○	○	○

*** 3. Professional literature (ie: journals & textbooks) and research findings are useful in my day-to-day practice**

Strongly Disagree	Disagree	Neutral	Agree	Strongly Agree
○	○	○	○	○

*** 4. I am interested in learning or improving the skills necessary to incorporate evidence-based practice into my osteopathic practice**

Strongly Disagree	Disagree	Neutral	Agree	Strongly Agree
○	○	○	○	○

* 5. Evidence-based practice improves the quality of my patient's care

Strongly Disagree	Disagree	Neutral	Agree	Strongly Agree
○	○	○	○	○

* 6. Evidence-based practice assists me in making decisions about patient care

Strongly Disagree	Disagree	Neutral	Agree	Strongly Agree
○	○	○	○	○

EBASE

INNOVATE-UK: International Osteopathy and Evidence-Based Practice Project

PART A: Beliefs (continued)

Please continue to rate your opinion on the following statements about evidence-based practice

* 7. Evidence-based practice takes into account my clinical experience when making clinical decisions

Strongly Disagree	Disagree	Neutral	Agree	Strongly Agree
○	○	○	○	○

* 8. Evidence-based practice takes into account a patient’s preference for treatment

Strongly Disagree	Disagree	Neutral	Agree	Strongly Agree
○	○	○	○	○

* 9. The adoption of evidence-based practice places an unreasonable demand on my practice

Strongly Disagree	Disagree	Neutral	Agree	Strongly Agree
○	○	○	○	○

* 10. There is a lack of evidence from clinical trials to support most of the treatments I use in my practice

Strongly Disagree	Disagree	Neutral	Agree	Strongly Agree
○	○	○	○	○

* 11. Prioritizing evidence-based practice within osteopathic practice is fundamental to the advancement of the profession

Strongly Disagree	Disagree	Neutral	Agree	Strongly Agree
○	○	○	○	○

PART B: Skills

Part B of the survey asks you to rate your current level of skill in several areas relating to evidence-based practice; the 5-point scale ranges from low to high.

*** 12. Identifying knowledge gaps in practice**

	Low	Low-medium	Medium	Medium-high	High
Level of skill:	◯	◯	◯	◯	◯

*** 13. Identifying answerable clinical questions**

	Low	Low-medium	Medium	Medium-high	High
Level of skill:	◯	◯	◯	◯	◯

*** 14. Locating professional literature (i.e. journal articles)**

	Low	Low-medium	Medium	Medium-high	High
Level of skill:	◯	◯	◯	◯	◯

*** 15. Online database searching (e.g. PubMed)**

	Low	Low-medium	Medium	Medium-high	High
Level of skill:	◯	◯	◯	◯	◯

*** 16. Retrieving evidence**

	Low	Low-medium	Medium	Medium-high	High
Level of skill:	◯	◯	◯	◯	◯

INNOVATE-UK: International Osteopathy and Evidence-Based Practice Project

PART B: Skills (continued)

Please continue to rate your current level of skill in the following areas relating to evidence-based practice.

*** 17. Critical appraisal of evidence**

	Low	Low-medium	Medium	Medium-high	High
Level of skill:	◯	◯	◯	◯	◯

*** 18. Synthesis of research evidence**

	Low	Low-medium	Medium	Medium-high	High
Level of skill:	○	○	○	○	○

*** 19. Applying research evidence to patient cases**

	Low	Low-medium	Medium	Medium-high	High
Level of skill:	○	○	○	○	○

*** 20. Sharing evidence with colleagues**

	Low	Low-medium	Medium	Medium-high	High
Level of skill:	○	○	○	○	○

*** 21. Conducting clinical research (e.g. clinical trials)**

	Low	Low-medium	Medium	Medium-high	High
Level of skill:	○	○	○	○	○

PART B: Skills (continued)

Please continue to rate your current level of skill in the following areas relating to evidence-based practice.

*** 22. Using findings from clinical research**

	Low	Low-medium	Medium	Medium-high	High
Level of skill:	○	○	○	○	○

*** 23. Conducting systematic reviews**

	Low	Low-medium	Medium	Medium-high	High
Level of skill:	○	○	○	○	○

*** 24. Using findings from systematic reviews**

	Low	Low-medium	Medium	Medium-high	High
Level of skill:	○	○	○	○	○

PART C: Training

Part C of the survey asks you to indicate the highest level of training/education you have received across several areas relating to evidence-based practice.

*** 25. Evidence-based practice / evidence-based osteopathy**

- None
- Seminar (less than 1 day)
- Short course (less than 1 week)
- Specific course (1 week or longer)
- Certificate
- Diploma
- Minor component of a study program
- Major component of a study program
- Other

If you selected other, please specify

*** 26. Applying research evidence to clinical practice**

- None
- Seminar (less than 1 day)
- Short course (less than 1 week)
- Specific course (1 week or longer)
- Certificate
- Diploma
- Minor component of a study program
- Major component of a study program
- Other

If you selected other, please specify

*** 27. Conducting clinical research (i.e. clinical trials)**

- None
- Seminar (less than 1 day)
- Short course (less than 1 week)
- Specific course (1 week or longer)
- Certificate
- Diploma
- Minor component of a study program
- Major component of a study program
- Other

If you selected other, please specify

*** 28. Conducting systematic reviews or meta-analysis (i.e. statistical analysis of data combined from two or more studies)**

- ◯ None
- ◯ Seminar (less than 1 day)
- ◯ Short course (less than 1 week)
- ◯ Specific course (1 week or longer)
- ◯ Certificate
- ◯ Diploma
- ◯ Minor component of a study program
- ◯ Major component of a study program
- ◯ Other

If you selected other, please specify

*** 29. Critical thinking / critical analysis**

- ◯ None
- ◯ Seminar (less than 1 day)
- ◯ Short course (less than 1 week)
- ◯ Specific course (1 week or longer)
- ◯ Certificate
- ◯ Diploma
- ◯ Minor component of a study program
- ◯ Major component of a study program
- ◯ Other

If you selected other, please specify

EBASE

INNOVATE-UK: International Osteopathy and Evidence-Based Practice Project

PART D: Use

Part D of the survey asks you indicate how often you have performed certain evidence-based practice related activities over the last month.

*** 30. I have read/reviewed professional literature (i.e. professional journals & textbooks) related to my practice**

	0 articles	1-5 articles	6-10 articles	11-15 articles	16 + articles
Over the last month:	◯	◯	◯	◯	◯

*** 31. I have read/reviewed clinical research findings related to my practice**

	0 articles	1-5 articles	6-10 articles	11-15 articles	16 + articles
Over the last month:	◯	◯	◯	◯	◯

*** 32. I have used professional literature or research findings to assist my clinical decision-making**

	Never	1-5 times	6-10 times	11-15 times	16 + times
Over the last month:	○	○	○	○	○

*** 33. I have used professional literature or research findings to change my clinical practice**

	Never	1-5 times	6-10 times	11-15 times	16 + times
Over the last month:	○	○	○	○	○

EBASE

INNOVATE-UK: International Osteopathy and Evidence-Based Practice Project

PART D: Use (continued)

Please continue to indicate how often you have performed certain evidence-based practice related activities over the last month.

*** 34. I have used an online database (e.g. PubMed, MEDLINE) to search for practice related literature or research**

	Never	1-5 times	6-10 times	11-15 times	16 + times
Over the last month:	○	○	○	○	○

*** 35. I have used an online search engine (e.g. Google) to search for practice related literature or research**

	Never	1-5 times	6-10 times	11-15 times	16 + times
Over the last month:	○	○	○	○	○

*** 36. I have consulted a colleague or industry expert to assist my clinical decision-making**

	Never	1-5 times	6-10 times	11-15 times	16 + times
Over the last month:	○	○	○	○	○

*** 37. I have referred to magazines, layperson/self-help books, or non-government/non-education institution websites to assist my clinical decision-making**

	Never	1-5 times	6-10 times	11-15 times	16 + times
Over the last month:	○	○	○	○	○

EBASE

INNOVATE-UK: International Osteopathy and Evidence-Based Practice Project

PART D: Use (continued)

Please answer the following questions about your use of evidence-based practice as indicated.

*** 38. What percentage of your practice do you estimate is based on clinical research evidence (i.e. evidence from clinical trials)?**

- None (0%)
- Very small proportion (1-25%)
- Small proportion (26-50%)
- Moderate proportion (51-75%)
- Large proportion (76-99%)
- All (100%)

*** 39. When you are making clinical decisions, in what order do the following sources of information inform the basis of your decision?** *(Please rank the items from 1 to 10, with 1 being the most frequently used source of information, to 10 being the least frequently used source of information)*

- Published clinical evidence (i.e. clinical trials)
- Published experimental/laboratory evidence (i.e. animal or test tube studies)
- Traditional knowledge
- Consulting fellow practitioners or experts
- Personal intuition
- Trial and error
- Textbooks
- Clinical practice guidelines
- Patient preference
- Personal preference

EBASE

INNOVATE-UK: International Osteopathy and Evidence-Based Practice Project

PART E: Barriers

Part E of the survey asks you to indicate to what extent certain factors prevent you from participating in evidence-based practice; the scale ranges from ‘not a barrier’ to ‘major barrier’.

*** 40. Lack of time**

Not a barrier	A minor barrier	A moderate barrier	A major barrier
○	○	○	○

*** 41. Lack of resources (i.e. access to a computer, the internet or online databases)**

Not a barrier	A minor barrier	A moderate barrier	A major barrier
○	○	○	○

*** 42. Lack of clinical evidence in osteopathy**

Not a barrier	A minor barrier	A moderate barrier	A major barrier
○	○	○	○

*** 43. Insufficient skills for locating research**

Not a barrier	A minor barrier	A moderate barrier	A major barrier
○	○	○	○

*** 44. Insufficient skills for interpreting research**

Not a barrier	A minor barrier	A moderate barrier	A major barrier
○	○	○	○

EBASE

INNOVATE-UK: International Osteopathy and Evidence-Based Practice Project

PART E: Barriers (continued)

Please continue to indicate to what extent these factors prevent you from participating in evidence-based practice

*** 45. Insufficient skills to critically appraise / evaluate the literature**

Not a barrier	A minor barrier	A moderate barrier	A major barrier
○	○	○	○

*** 46. Insufficient skills to apply research findings to clinical practice**

Not a barrier	A minor barrier	A moderate barrier	A major barrier
○	○	○	○

*** 47. Lack of incentive to participate in evidence-based practice**

Not a barrier	A minor barrier	A moderate barrier	A major barrier
○	○	○	○

*** 48. Lack of interest in evidence-based practice**

Not a barrier	A minor barrier	A moderate barrier	A major barrier
○	○	○	○

EBASE

INNOVATE-UK: International Osteopathy and Evidence-Based Practice Project

PART E: Barriers (continued)

Please continue to indicate to what extent these factors prevent you from participating in evidence-based practice

*** 49. Lack of relevance to osteopathy practice**

Not a barrier	A minor barrier	A moderate barrier	A major barrier
○	○	○	○

*** 50. Lack of colleague support for evidence-based practice**

Not a barrier	A minor barrier	A moderate barrier	A major barrier
○	○	○	○

*** 51. Lack of industry support for evidence-based practice**

Not a barrier	A minor barrier	A moderate barrier	A major barrier
○	○	○	○

*** 52. Patient preference for treatment**

Not a barrier	A minor barrier	A moderate barrier	A major barrier
○	○	○	○

EBASE

INNOVATE-UK: International Osteopathy and Evidence-Based Practice Project

PART F: Facilitators

Part F of the survey asks you indicate to what extent certain strategies assist you in participating in evidence-based practice; the scale ranges from ‘not useful’ to ‘very useful’.

*** 53. Access to the Internet in your workplace**

Not useful	Slightly useful	Moderately useful	Very useful
○	○	○	○

*** 54. Access to free online databases in the workplace, such as Cochrane and PubMed**

Not useful	Slightly useful	Moderately useful	Very useful
○	○	○	○

*** 55. Free access to online databases that usually require license fees, such as MEDLINE and CINAHL**

Not useful	Slightly useful	Moderately useful	Very useful
○	○	○	○

*** 56. Ability to download full-text / full-length journal articles**

Not useful	Slightly useful	Moderately useful	Very useful
○	○	○	○

*** 57. Access to online education materials related to evidence based practice**

Not useful	Slightly useful	Moderately useful	Very useful
○	○	○	○

PART F: Facilitators (continued)

Please continue to indicate to what extent these factors assist you in participating in evidence-based practice

*** 58. Access to tools used to assist the critical appraisal / evaluation of research evidence**

Not useful	Slightly useful	Moderately useful	Very useful
○	○	○	○

*** 59. Access to critically appraised topics relevant to osteopathy (these are critical appraisals of single research papers)**

Not useful	Slightly useful	Moderately useful	Very useful
○	○	○	○

* **60. Access to critical reviews of research evidence relevant to osteopathy (these are critical reviews of multiple research papers addressing a single topic)**

Not useful	Slightly useful	Moderately useful	Very useful
○	○	○	○

* **61. Access to research rating tools that facilitate critical appraisal of single research papers**

Not useful	Slightly useful	Moderately useful	Very useful
○	○	○	○

* **62. Access to online tools that assist you to conduct your own critical appraisals of multiple research papers related to a single topic**

Not useful	Slightly useful	Moderately useful	Very useful
○	○	○	○

EBASE

INNOVATE-UK: International Osteopathy and Evidence-Based Practice Project

PART G: Demographics

The final part of this survey asks you about your age, sex, qualifications and health-related work experience.

* **63. Which category below includes your age?**

- ○ 19 years or under
- ○ 20-29 years
- ○ 30-39 years
- ○ 40-49 years
- ○ 50-59 years
- ○ 60-69 years
- ○ 70 years or older

* **64. What is your gender?**

- ○ Male
- ○ Female
- ○ Other

*** 65. What is your highest qualification?**

- ◯ High School Certificate
- ◯ Certificate
- ◯ Diploma
- ◯ Advanced Diploma
- ◯ Bachelor Degree
- ◯ Honours Degree
- ◯ Graduate Certificate / Diploma
- ◯ Masters Degree
- ◯ PhD / Doctorate

Other (please specify)

[ ]

PART G: Demographics (continued)

*** 66. How many years ago did you receive your highest qualification?**

- ◯ Less than 1 year
- ◯ 1-5 years
- ◯ 6-10 years
- ◯ 11-15 years
- ◯ 16 years or over

*** 67. How many years have you practiced osteopathy?**

- ◯ Less than 1 year
- ◯ 1-5 years
- ◯ 6-10 years
- ◯ 11-15 years
- ◯ 16 years or more

*** 68. How many hours per week do you typically practice as an osteopath in a clinical setting?**

- 0 (none)
- 1-5 hours
- 6-10 hours
- 11-15 hours
- 16-20 hours
- 21-25 hours
- 26-30 hours
- 31-35 hours
- 36-40 hours
- 41-45 hours
- 46-50 hours
- 50+ hours

PART G: Demographics (continued)

*** 69. How many hours per week do you typically participate in research?**

- 0 (none)
- 1-5 hours
- 6-10 hours
- 11-15 hours
- 16-20 hours
- 21-25 hours
- 26-30 hours
- 31-35 hours
- 36-40 hours
- 41-45 hours
- 46-50 hours
- 50+ hours

*** 70. How many hours per week do you typically teach in the higher education sector?**

- 0 (none)
- 1-5 hours
- 6-10 hours
- 11-15 hours
- 16-20 hours
- 21-25 hours
- 26-30 hours
- 31-35 hours
- 36-40 hours
- 41-45 hours
- 46-50 hours
- 50+ hours

EBASE

INNOVATE-UK: International Osteopathy and Evidence-Based Practice Project

PART G: Demographics (continued)

*** 71. Which types of treatment / management do you typically provide in your first consultation as an osteopath? (please tick all that apply)**

- [ ] Articulation
- [ ] Acupuncture / acupressure
- [ ] Cranial technique
- [ ] Electrotherapy
- [ ] Exercise
- [ ] Functional technique
- [ ] General osteopathic treatment
- [ ] HVLA thrust
- [ ] Ice / cold treatment
- [ ] Muscle energy therapy
- [ ] Myofascial release (MFR)
- [ ] Orthotics
- [ ] Relaxation advice
- [ ] Soft tissue therapy
- [ ] Strain-counterstrain
- [ ] Steroid injection
- [ ] Visceral therapy
- [ ] Other (please specify)

EBASE

INNOVATE-UK: International Osteopathy and Evidence-Based Practice Project

PART G: Demographics (continued)

*** 72. In what setting do you practice osteopathy predominantly?**

- ◯ Solo practice
- ◯ With a group of conventional medical (e.g. general practitioner) and allied health (e.g. physiotherapist) practitioners
- ◯ With a group of complementary and alternative medicine (CAM) practitioners
- ◯ With a combination of CAM and conventional medical and allied health practitioners
- ◯ Within a clinical institution (e.g. hospital, nursing home)
- ◯ Within an educational institution (e.g. university)

*** 73. In which region of the UK do you practice osteopathy? (If you practice across multiple regions, please select the one that you practice in predominantly)**

- ◯ East Anglia
- ◯ London (UK)
- ◯ Midlands (UK)
- ◯ Northern Ireland
- ◯ North West UK
- ◯ North East UK
- ◯ Scotland
- ◯ South East UK
- ◯ South West UK
- ◯ Wales
- ◯ Other (please specify)

[ ]

*** 74. Which osteopathy professional associations are you a member of?**

- ◯ Foundation for Paediatric Osteopathy
- ◯ Institute of Classical Osteopathy (ICO)
- ◯ Institute of Osteopathy (iO)
- ◯ Molinari Institute Of Health
- ◯ Rollin E Becker Institute
- ◯ Society of Osteopaths in Animal Practice
- ◯ Sutherland Cranial College of Osteopathy
- ◯ Sutherland Society
- ◯ Other (please specify)

[ ]

PART G: Demographics (continued)

*** 75. In what geographical region do you practice osteopathy? (If you practice across several geographical regions, please select the one region that you practice in predominantly)**

- ◯ City (Central business district)
- ◯ Inner city suburbs
- ◯ Outer city suburbs
- ◯ Rural / remote region

76. Are there any final comments you would like to add about evidence-based practice?

Thank you immensely for your participation in this survey - we are most grateful for your time and contribution.
